# The MATRICS Consensus Cognitive Battery: Psychometric Properties of the Chinese Version in Young Patients With Major Depression Disorder

**DOI:** 10.3389/fpsyt.2021.745486

**Published:** 2021-10-28

**Authors:** Sixiang Liang, Xiaomeng Xing, Mingwan Wang, Dan Wei, Tengfei Tian, Jun Liu, Sha Sha

**Affiliations:** ^1^The National Clinical Research Center for Mental Disorders, Beijing Key Laboratory of Mental Disorders, Beijing Anding Hospital, Capital Medical University, Beijing, China; ^2^The Advanced Innovation Center for Human Brain Protection, Capital Medical University, Beijing, China; ^3^School of Nursing, Peking Union Medical College, Beijing, China

**Keywords:** MCCB, adolescent, major depressive disorder, cognitive impairment, young, psychometric properties

## Abstract

**Background:** Young patients with major depressive disorder are also associated with cognitive deficits. The development of an accurate and effective battery to measure cognitive impairment in young patients with major depressive disorder (Y-MDD) is necessary for both research and clinical practice. This study was designed to test the psychometric properties of the Measurement and Treatment Research to Improve Cognition in Schizophrenia (MATRICS) Consensus Cognitive Battery (MCCB) in Y-MDD.

**Method:** Fifty Y-MDD patients, 38 euthymic young patients with bipolar disorder (Y-BD), and 51 healthy teenagers were recruited. The MCCB and the Montreal Cognitive Assessment (MoCA) were administered to assess cognitive impairment at baseline. The MCCB was also assessed 2 weeks later in Y-MDD patients. All subjects were between the ages of 13 and 24 years.

**Result:** In the current study, cognitive impairment was greater in Y-BD patients than in Y-MDD patients in some domains. The MCCB has good internal consistency and reliability in Y-MDD patients. The Pearson correlation coefficients for retest reliability were good. Our findings also revealed an acceptable correlation between the MCCB and the MoCA, indicating good concurrent validity of the MCCB. Furthermore, exploratory factor analysis of the MCCB in Y-MDD patients revealed five domains with acceptable internal structures.

**Conclusion:** The MCCB has acceptable psychometric properties and is a sensitive battery of cognitive impairment in Y-MDD patients. In the future, additional studies need to be carried out with larger samples while controlling for the use of psychotropic medications and antidepressants to validate the findings of the present study.

## Introduction

Cognitive impairment is a consistent feature in patients with major depressive disorder (MDD) and has attracted increasing attention from researchers and in clinical practice (1, 2). Previous studies also demonstrated that cognitive deficits may persist over time, even during remission of clinical symptoms (3) and may significantly impact functional recovery and treatment compliance (4). There are several standardized scales that measure cognitive function in MDD patients, such as the Controlled Oral Word Association (COWA) test (5), the Wisconsin Card Sorting Test (WCST) (6), and the Stroop Color and Word Test (7). However, the scales mentioned above could not comprehensively evaluate the patient's cognitive impairments. Therefore, some current studies have applied systematic batteries of tests to assess cognitive impairment in MDD patients (8–10). Researchers have also noted different cognitive patterns and profiles in different psychiatric disorders (11, 12). MDD is correlated with marked cognitive dysfunction, with effects in the medium to large range across a series of tests for cognitive impairment (8, 13). Identifying the latent structure of cognition in MDD has important implications for clinical practice and research. Applying factor analysis provides differing conceptualizations about the separability of the causal factors in cognitive dysfunction. The exploration of various structures for cognition in MDD has important implications for the development of potential treatments to improve cognitive function (14).

The National Institute of Mental Health (NIMH) of the United States has launched an initiative named the Measurement and Treatment Research to Improve Cognition in Schizophrenia (MATRICS). The MATRICS Consensus Cognitive Battery (MCCB) developed by NIMH aimed to assess cognitive deficits in schizophrenia (15). The MCCB is a comprehensive measurement including seven cognitive domains covering a wide range of neurocognitive functions, and it has high reliability and validity in schizophrenia (16). Several studies have used the MCCB to evaluate the level of cognition in MDD patients, and it has achieved great success (17–19). Our previous research also found good psychometric properties and revealed four cognitive factors when applying the MCCB to Chinese adult MDD patients (13).

Unfortunately, compared with those in adult MDD, efficient and comprehensive instruments for cognitive assessment in young patients with MDD (Y-MDD) have rarely been explored. The phenomenon of cognitive impairment in Y-MDD is prominent (20). The World Health Organization (WHO) states that Y-MDD is a leading cause of disability (21). Recent studies have shown that early onset of this illness could adversely disrupt educational achievement, interpersonal functioning, and vocational engagement (22, 23). Furthermore, cognitive impairment is also recognized as a common feature in Y-MDD, with a rate as high as 83% (24). However, cognitive symptoms in Y-MDD have seldom been explored in clinical practice and research efficiently and satisfactorily (20). Therefore, a previous review concluded that clinical observations on cognitive dysfunction in younger age groups are very important for clinical prevention (25).

A useful battery that can provide comprehensive details about neurocognitive functioning in Y-MDD is crucial. With a well-established battery, exploring the cognitive structure will also contribute to the development of potential treatments to improve cognitive function in Y-MDD patients. In summary, providing a comprehensive evaluation of the psychometric properties of the MCCB in Y-MDD patients could satisfactorily meet the needs mentioned above. The researchers hypothesized that the MCCB is a good assessment of cognitive impairment in Y-MDD patients. Furthermore, with the MCCB, the cognitive structure could be clarified in Y-MDD patients.

## Materials and Methods

### Participants

Fifty Y-MDD patients, 38 euthymic young patients with bipolar disorder (Y-BD), and 51 healthy controls were included. This research was conducted in Beijing Anding Hospital (a tertiary hospital for psychiatric disorders in Beijing, China). All patients were recruited from February 2021 to June 2021 at the Department of Major Depressive Disorder, Beijing Anding Hospital. All patients met the diagnostic criteria according to the International Statistical Classification of Diseases and Related Health Problems 10th Revision (ICD-10) and were diagnosed by an experienced psychiatrist. Meanwhile, patients with severe physical diseases, alcoholics, and drug addicts were excluded from the current study. Simultaneously, adolescent patients with other cognitive disorders, such as intellectual disability and autism spectrum disorder, were also excluded. Some medications for adolescent patients, such as antidepressants, mood stabilizers, and atypical antipsychotics, were allowed during the study. Healthy adolescents matched by age, sex, and level of education were recruited via general advertisements in the school and community. Written informed consent was obtained from all enrolled participants. The study protocol was also approved by the Ethics Committee of the Beijing Anding Hospital, Capital Medical University (approval protocol number: 2020 scientific research no. 16).

### Assessment Procedure

Clinical variables and sociodemographic information were collected in this study. The severity of the affective symptoms was determined using the Hamilton Depression Rating Scale-17 (HDRS-17) and the Young Mania Rating Scale (YMRS) by one professional psychiatrist. The level of cognition was measured by the MCCB for all participants. Meanwhile, Y-MDD patients were assessed for MCCB at baseline and 2 weeks later. The MoCA was also measured for Y-MDD patients at baseline. The other patients and healthy controls were only assessed with the MCCB. Meanwhile, euthymic Y-BD patients were defined as having an HDRS-17 score ≤ 8 and a YMRS score ≤ 6, and all euthymic Y-BD patients were in clinical remission for at least 1 month. MDD patients were defined as having an HDRS total score ≥18. All of these definitions follow those defined in a previous study (16).

In this study, we used the authorized MCCB (Chinese version), which was introduced to China by the Mental Health Institute of Peking University. In our recent research, we demonstrated that the MCCB had good psychometric characteristics in adult patients with MDD (16). The Chinese version of the MCCB contains nine items in seven domains. Higher scores on all items reflect better neurocognitive function in the corresponding domains. The scores after adjusting for age, sex, and educational levels (T-score) for each item and domain were calculated in this study, and lower scores indicate more severe cognitive impairment. The details of the items and domains are as follows:

- Speed of processing (SOP): (a) Trail Making Test, Part A (TMT-A); (b) Brief Assessment of Cognition in Schizophrenia, Symbol Coding subtest (BACS-SC); and (c) Category Fluency: animal naming (Fluency);
- Verbal learning (VRB): Hopkins Verbal Learning Test—Revised (HVLT-R);
- Visual learning (VIS): Brief Visuospatial Memory Test —Revised (BVMT-R);
- Reasoning and problem solving (RPS): Neuropsychology Assessment Battery, Mazes (NAB-Mazes);
- Working memory (WM): Wechsler Memory Scale, third edition, Spatial Span (WMS-III SS);
- Attention/vigilance (AV): Continuous Performance Test, Identical Pairs version (CPT-IP); and
- Social cognition (SC): Mayer–Salovey–Caruso Emotional Intelligence Test, Managing Emotions (MSCEIT ME).

The item Letter–Number Span (LNS) has no corresponding structure in Chinese. It has been deleted in the Chinese version of the MCCB (15). Considering the concurrent validity, we also assessed the MoCA in the patients with MDD at baseline. The MoCA, as a widely used scale, was applied to assess cognitive deficits in the community. A previous study also demonstrated that the MoCA has excellent psychometric properties for distinguishing mild cognitive impairment between patients and the healthy population (17). The MoCA is a scale covering seven domains: execution function, visual–spatial ability, attention and concentration, abstraction, fluency, delayed memory, and orientation (18).

### Statistical Analysis

Data analysis in the current study was performed using SPSS software. Multifactor analyses of variance and chi-square tests were used to compare the continuous variables and categorical variables, respectively. The internal consistency of the MCCB was tested using Cronbach's alpha. Internal reliability was defined as acceptable when Cronbach's alpha was >0.60 (26). Test–retest reliability and concurrent validity were explored using Pearson's correlation coefficients. The group cognitive differences were tested by analysis of covariance (ANCOVA) comparing the T-scores for each domain in the MCCB among the three groups. Exploratory factor analysis (EFA) with varimax rotation was applied to explore the internal structure of the MCCB for Y-MDD patients. The coefficients of each item ≥0.5 were defined as “acceptable” (27).

## Results

### Demographic Characteristics

In this study, 50 Y-MDD patients (16 males and 34 females), 38 Y-BD patients (18 males and 20 females), and 51 healthy controls (20 males and 31 females) completed the baseline assessment. In addition, 29 Y-MDD patients completed the second assessment. However, 21 Y-MDD patients failed to complete the retest 2 weeks later. No differences in age, education years, or sex were found among the three groups. On the other hand, there were no significant differences in the duration, the duration of this episode, or the timing of the episodes between the Y-MDD and Y-BD groups. We also compared the demographic characteristics and the HDRS scores between the Y-MDD patients who completed the second assessment (Y-MDDc) and the Y-MDD patients who failed to complete the retest (Y-MDDf). There was no significant difference between the two subgroups (Table 1).

**Table 1:** Demographic characteristics of each group.

	**Y-MDD**			**Y-BD**	**HC**	***p*-value**
	**All (*n* = 50)**	**Y-MDDc (*n* = 29)**	**Y-MDDf (*n* = 21)**	**(*n* = 38)**	**(*n* = 51)**	
Age (years)^+^	17.9 (2.7)	17.6 (2.7)	18.2 (2.6)	18.9 (3.0)	17.7 (3.2)	0.128
Sex (n, %)						0.346
Female	34 (66)	21 (72.4)	13 (61.9)	20 (52.6)	31 (60)	
Male	16 (33)	8 (27.6)	8 (38.1)	18 (47.4)	20 (40)	
Education years^+^	11.4 (2.2)	11.0 (2.7)	12.0 (2.0)	12.8 (2.8)	11.6 (2.8)	0.051
Duration (months)^+^	35.0 (35.8)	40.2 (36.7)	27.6 (34.1)	39.2 (31.2)	-	
The duration of this episode^+^	5.3 (7.8)	5.0 (8.8)	5.9 (6.1)	4.7 (8.8)	-	
Time of episodes^+^	2.2 (1.3)	2.4 (1.4)	1.9 (1.2)	2.6 (1.6)	-	
The score of HDRS^+^	22.2 (3.2)	22.6 (2.9)	21.6 (3.6)	3.4 (2.8)	-	
The score of YMRS	NA	NA	NA	1.8 (2.4)		
*T*-score of each domain^+^						*F*(*df* = 2)
Speed of processing	35.3 (12.6)	36.4 (11.7)	32.9 (12.9)	28.3 (13.7)	43.8 (10.1)	6.90**
Attention/vigilance	37.2 (13.0)	37.7 (12.6)	35.4 (13.9)	28.4 (14.2)	38.6 (12.7)	4.49*
Working memory	44.2 (12.8)	43.5 (12.9)	43.0 (13.3)	36.2 (8.4)	45.4 (10.0)	3.96^*^
Verbal learning	44.6 (9.2)	45.5 (8.4)	41.7 (10.0)	39.4 (9.3)	52.2 (11.5)	6.46^**^
Visual learning	42.0 (13.4)	41.9 (14.1)	41.6 (12.1)	40.1 (10.7)	47.4 (10.7)	5.54^**^
Reasoning and problem solving	43.2 (10.9)	41.4 (11.1)	44.1 (10.6)	35.6 (7.6)	49.3 (8.9)	8.48^**^
Social cognition	46.7 (15.3)	46.4 (13.1)	49.2 (17.5)	41.3 (15.2)	44.3 (10.2)	1.84

*Y-MDD, young patients with major depressive disorders; Y-MDDc, Y-MDD patients who completed the second assessment; Y-MDDf, Y-MDD patients who failed to complete the retest; Y-BD, young patients with bipolar disorder in the euthymic phase; HC, healthy controls; p-value, the p-value by analysis of variance or analysis of covariance (ANCOVA) among Y-MDD, Y-BD, and HC.*

+ *Mean (SD);*

* *p < 0.005;*

** *p < 0.001*.

**Figure 1:**
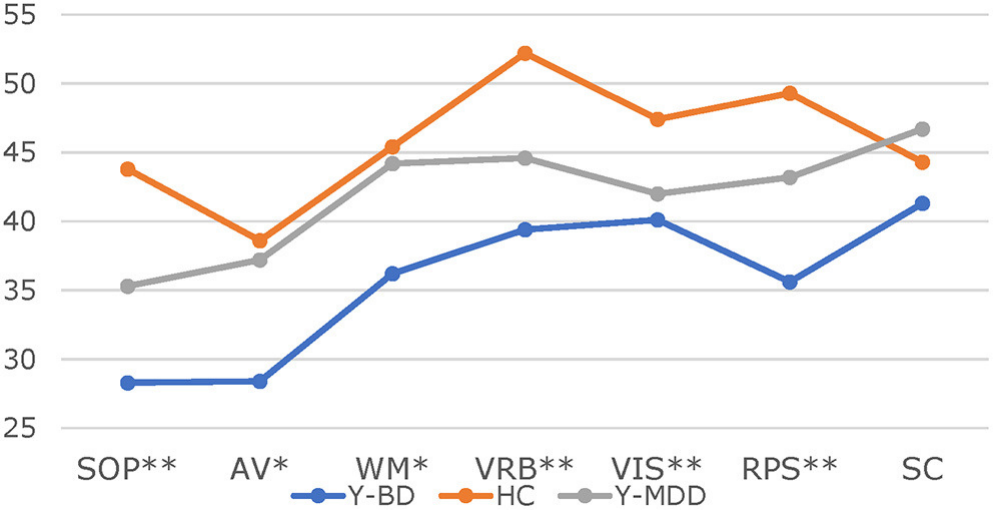
*T*-scores in the seven domains between different groups. * < 0.005, ** < 0.001. SOP, Speed of processing; AV, attention/vigilance; WM, working memory; VRB, verbal learning; VIS, visual learning; RPS, reasoning and problem solving; SC, social cognition.

The cognitive differences assessed by the MCCB between Y-MDD patients (*n* = 50), Y-BD patients (*n* = 38), and healthy controls (*n* = 51) were analyzed depending on the baseline data. After adjustment for age, sex, and education years, there were significant differences in every domain (except for SC) among the three groups, such as SOP (*F* = 6.90, *p* < 0.001), AV (*F* = 4.49, *p* < 0.001), WM (*F* = 3.96, *p* < 0.01), VRB (*F* = 6.46, *p* < 0.001), VIS (*F* = 5.54, *p* < 0.001), RPS (*F* = 8.48, *p* < 0.001), and SC (*F* = 1.84, *p* = 0.1). We also found that cognitive impairment was more severe in Y-BD patients than in Y-MDD patients in some domains. The details of the significantly different domains mentioned above after adjusting for age, sex, and education years are shown as follows: AV (*F* = 2.94, *p* < 0.05), WM (*F* = 2.55, *p* < 0.05), and RPS (*F* = 3.11, *p* < 0.05). Furthermore, after adjusting for age, sex, and education years, we compared the differences in cognitive impairment between Y-MDD patients and healthy controls. As we expected, significant differences were found in most domains, except for WM (*F* = 0.73, *p* = 0.576) and RPS (*F* = 2.42, *p* = 0.05). The same method was used to explore the differences in cognitive impairment between Y-BD patients and healthy controls. Significant differences were found in almost all domains, except for SC (*F* = 1.06, *p* = 0.38). The details are as follows: SOP (*F* = 7.69, *p* < 0.001), AV (*F* = 3.60, *p* < 0.01), WM (*F* = 5.40, *p* < 0.005), VRB (*F* = 7.27, *p* < 0.001), VIS (*F* = 5.22, *p* < 0.005), and RPS (*F* = 12.73, *p* < 0.001) (Table 1, Figure 1).

### Reliability Test

For the internal consistency analysis, Cronbach's alpha of the MCCB was 0.79 at baseline and 0.83 2 weeks later. In addition, Cronbach's alpha, if each item was deleted from the nine items of the MCCB, ranged from 0.75 to 0.81 at baseline and 0.77 to 0.86 2 weeks later. Cronbach's alphas in the domains of AV, VIS, VRB, and SOP ranged from 0.60 to 0.87 at baseline and from 0.58 to 0.91 2 weeks later. We did not explore Cronbach's alpha for three domains (SC, RPS, and WM) because there was only one measure in those domains (Table 2).

**Table 2:** The internal consistency reliability in Y-MDD patients.

**Domains**	**Measures (*n* = 9)**	**Alpha if item deleted (Week 0)**	**Cronbach's alpha (Week 0)**	**Alpha if item deleted (Week 2)**	**Cronbach's alpha (Week 2)**
	Total		0.79		0.83
Speed of processing			0.61		0.58
	TMT-A	0.75		0.80	
	BACS-SC	0.75		0.81	
	Fluency	0.77		0.81	
Verbal learning			0.83		0.87
	HVLT-R	0.76		0.83	
Visual learning			0.86		0.84
	BVMT-R	0.77		0.81	
Attention/vigilance			0.87		0.90
	CPT-IP	0.75		0.80	
Working memory					
	WMS-III SS	0.76		0.80	
Reasoning and problem solving					
	NAB Mazes	0.77		0.78	
Social cognition					
	MSCEIT ME	0.81		0.86	

*TMT-A, Trail Making Test, Part A; BACS-SC, Brief Assessment of Cognition in Schizophrenia: Symbol Coding; Fluency, Category Fluency: Animal Naming; HVLT-R, Hopkins Verbal Learning Test—Revised; BVMT-R, Brief Visuospatial Memory Test—Revised; CPT-IP, Continuous Performance Test—Identical Pairs; WMS-III SS, Wechsler Memory Scale-III Spatial Span; NAB-Mazes, Neuropsychological Assessment Battery, Mazes; MSCEIT ME, Mayer–Salovey–Caruso Emotional Intelligence Test, Managing Emotions*.

For the retest reliability, 29 Y-MDD patients completed the second assessment of the MCCB 2 weeks later. There was a significant correlation in most domains. The Pearson correlation coefficients of the seven domains were 0.71 in AV, 0.70 in SC, 0.76 in WM, 0.81 in RPS, 0.74 in SOP, and 0.59 in VIS (*p*-values < 0.01). Unfortunately, we did not find a significant correlation for the domain of VRB (*r* = 0.41, *p* = 0.05).

### Validity Test

The concurrent validity was tested by Pearson correlation analysis to evaluate the associations between the MoCA and the MCCB. The domain of recall in the MoCA was correlated with the *T*-scores of the SOP (*r* = 0.47, *p* < 0.05), RPS (*r* = 0.53, *p* < 0.01), and SC (*r* = 0.41, *p* < 0.05) at baseline.

EFA with varimax rotation was carried out to explore the internal structure of the MCCB depending on the baseline data. By means of varimax rotation, when the coefficients were equal to or < 0.5, they were omitted (KMO = 0.767, *p* < 0.001). Finally, five factors were obtained. The details of the five factors, VIS (Factor 1, with three items), AV (Factor 2, with three items), VRB (Factor 3, with three items), WM and RPS (Factor 4, with four items), and SC with BACS (Factor 5, with two items), cumulatively accounted for 74.8% of the variance. The details are shown in Table 3.

**Table 3:** EFA of MCCB in Y-MDD patients.

	**1**	**2**	**3**	**4**	**5**
TMT-A				−0.629	
BACS-SC					0.577
HVLT-R2			0.766		
HVLT-R1			0.868		
HVLT-R3			0.748		
WMS-III SS				0.722	
NAB-Mazes				0.857	
BVMT-R1	0.777				
BVMT-R2	0.929				
BVMT-R3	0.855				
Fluency				0.583	
MSCEIT ME					0.883
CPT-IP1		0.888			
CPT-IP2		0.830			
CPT-IP3		0.629			

*Coefficients lower than or equal to 0.50 were omitted.*

*NAB-Mazes, Neuropsychology Assessment Battery, Mazes subtest; BVMT-R, Brief Visuospatial Memory test—Revised (e.g., BVMT-1 means first test, BVMT-2 means second test); WMS-III SS, Wechsler Memory Scale, third edition, Spatial Span subtest; BACS-SC, Brief Assessment of Cognition in Schizophrenia; TMT-A, Trail-Making Test, Part A; CPT-IP, Continuous Performance Test—Identical Pairs version (CPT-1, two-digit test; CPT-2, three-digit test; CPT-3, four-digit test); HVLT-R, Hopkins Verbal Learning Test—Revised, Immergence recall (e.g., HVLT-1 means first test; HVLT-2 means second test); HVLT-R, Hopkins Verbal Learning Test—Emotional Intelligence, Category Fluency*.

## Discussion

To the best of our knowledge, this is the first study to explore the psychometric properties of the MCCB in adolescent MDD patients. The results demonstrated that the MCCB may be clinically useful as a cognitive defect rating battery for Y-MDD.

For cognitive differences, we assessed the cognitive functioning of MDD, BD, and healthy controls, aged 13–24 years. Similar to findings in previous studies, patients with affective disorder had a lower cognitive level than healthy controls (28, 29). In contrast to the findings from research focused on adult patients (30), cognitive differences were not prominent in our study. We deduced that patients exhibited greater cognitive impairment with increasing illness duration. Young patients with a short illness duration in the current study show no significant cognitive impairment. Previous research also supported our speculation (31). Even so, our study also found that cognitive impairment was worse among Y-BD patients than Y-MDD patients for some cognitive domains. These results are consistent with previous research (32).

In the current study, Cronbach's alphas for all items and domains (all but the speed of processing) were good at baseline and 2 weeks later. These results indicated that the MCCB had excellent internal consistency in Y-MDD patients. A similar result was also found in our previous research for adult patients with MDD (13). The current result supports the finding that the MCCB is a good choice when administered to Y-MDD and adult MDD patients. Although the internal consistency of the MCCB was excellent in our study, similar to our previous study, Cronbach's alpha was still lower than the coefficient of Cronbach's alpha (0.923) in schizophrenia (SCH), which was explored in the Chinese Norm Manual (Chinese edition) (33). We speculated that cognitive deficits have diverse features in different mental disorders (12). Compared with that for MDD, the sensitivity of detecting cognitive impairment by the MCCB might be more suitable for SCH. Meanwhile, for test–retest reliability, our results also showed an acceptable outcome. The MATRICS committee stated that if the *R* value is >0.7, the result is acceptable (15). Most coefficients were satisfactory except for VRB (*r* = 0.41) and VIS (*r* = 0.59). We deduced that the retest interval was not long enough, which could weaken this correlation in Y-MDD patients.

In the current study, the recall score on the MoCA was significantly and positively associated with the *T*-scores of parts of the domains of the MCCB. Compared with that of our previous study in adult MDD patients, the concurrent validity was not good (13). On the one hand, we considered the possibility that the sensitivity and discrimination of the MoCA to evaluate cognitive impairment in Y-MDD are insufficient. A previous study demonstrated that cognitive deficits are linked with the age of onset and duration (34). Compared with that in adult patients with MDD, the cognitive impairment in Y-MDD is less severe (35, 36). Meanwhile, the MoCA is used widely in the community to assess mildly impaired cognition (37). Therefore, we deduced that the unsatisfactory consistent validity was due to the mismatch between the degree of cognitive deficit and the validity criterion. On the other hand, in the current study, the retest sample size was small. An insufficient sample size might weaken the correlation of the validity criterion.

The EFA of the MCCB showed five dimensions in the current study, indicating an acceptable internal structure of the MCCB when used with Y-MDD patients. Unfortunately, we failed to reproduce the theoretical model of the MCCB, which aims to explore seven independent factors (33). The theoretical model was also found in McCleery et al.'s findings, which focused on SCH patients (14). However, compared to our previous study in adult MDD patients, the internal structure in Y-MDD patients was better. We noted that the MCCB was originally developed to assess cognitive dysfunction in SCH. Cognitive deficits have many differences between MDD and SCH (12). Different cognitive characteristics may lead to deviations in the internal structure of the MCCB (12, 38). Many recent studies also support our study and have been unable to reproduce the theoretical model (39, 40). Meanwhile, the LNS was not enrolled in the MCCB (Chinese version), and the absence of these items could have partly influenced the internal structure.

This is the first study to explore the psychometric properties of the MCCB in adolescent MDD patients. However, there are still some limitations in this study. First, the MoCA was originally designed for people with mild impaired cognition in the community, and its low specificity could have weakened the concurrent validity of the MCCB in Y-MDD patients. Second, we have no control over the use of psychotropic medications and antidepressants, which might bias the cognitive assessment (41). Third, the MoCA assessment was only applied in the Y-MDD group. The lack of a significance analysis of possible group differences may weaken the robustness of this study. Finally, the sample size was insufficient, which could impede the validity analysis of the MCCB. Considering previous studies, the number of participants was five times more than the number of items in the analysis, which is the frequently recommended approach when performing an EFA (42). Therefore, the sample size of each group for ~70 (rather than 50) is more suitable.

In summary, in the current study, the MCCB shows good psychometric properties for Y-MDD patients. Currently, the MCCB is applied as a comprehensive and acceptable battery for Y-MDD patients in clinical practice and research. However, in the future, other studies need to be carried out with larger samples while controlling for the use of psychotropic medications and antidepressants to validate the findings of the present study.

## Data Availability Statement

The original contributions presented in the study are included in the article/supplementary material, further inquiries can be directed to the corresponding author.

## Ethics Statement

The studies involving human participants were reviewed and approved by the Ethics Committee of the Beijing Anding Hospital, Capital Medical University. Written informed consent to participate in this study was provided by the participants' legal guardian/next of kin.

## Author Contributions

SL and XX: writing—original draft and writing—review and editing. MW: conceptualization. DW and TT: formal analysis and writing—review and editing. JL: formal analysis, methodology, and writing—review and editing. SS: data curation and writing—review and editing. All authors contributed to the article and approved the submitted version.

## Funding

This study was supported by the Beijing Municipal Administration of Hospitals Incubating Program (PX2018063) and the Beijing Anding Hospital, Capital Medical University (YJ2021-05).

## Conflict of Interest

The authors declare that the research was conducted in the absence of any commercial or financial relationships that could be construed as a potential conflict of interest.

## Publisher's Note

All claims expressed in this article are solely those of the authors and do not necessarily represent those of their affiliated organizations, or those of the publisher, the editors and the reviewers. Any product that may be evaluated in this article, or claim that may be made by its manufacturer, is not guaranteed or endorsed by the publisher.
